# Effects of S-Propargyl-Cysteine (SPRC) in Caerulein-Induced Acute Pancreatitis in Mice

**DOI:** 10.1371/journal.pone.0032574

**Published:** 2012-03-01

**Authors:** Jenab N. Sidhapuriwala, Akhil Hegde, Abel D. Ang, Yi Zhun Zhu, Madhav Bhatia

**Affiliations:** 1 Department of Pharmacology, National University of Singapore, Singapore, Singapore; 2 Department of Pathology, University of Otago, Christchurch, New Zealand; 3 Department of Pharmacology, Fudan University, Shanghai, China; University of Cincinnati, United States of America

## Abstract

Hydrogen sulfide (H_2_S), a novel gaseous messenger, is synthesized endogenously from L-cysteine by two pyridoxal-5′-phosphate-dependent enzymes, cystathionine β-synthase (CBS) and cystathionine γ-lyase (CSE). S-propargyl-cysteine (SPRC) is a slow H_2_S releasing drug that provides cysteine, a substrate of CSE. The present study was aimed to investigate the effects of SPRC in an *in vivo* model of acute pancreatitis (AP) in mice. AP was induced in mice by hourly caerulein injections (50 µg/kg) for 10 hours. Mice were treated with SPRC (10 mg/kg) or vehicle (distilled water). SPRC was administered either 12 h before or 3 h before the induction of pancreatitis. Mice were sacrificed 1 h after the last caerulein injection. Blood, pancreas and lung tissues were collected and processed to measure the plasma amylase, plasma H_2_S, myeloperoxidase (MPO) activities and cytokine levels in pancreas and lung. The results revealed that significant reduction of inflammation, both in pancreas and lung was associated with SPRC given 3 h prior to the induction of AP. Furthermore, the beneficial effects of SPRC were associated with reduction of pancreatic and pulmonary pro-inflammatory cytokines and increase of anti-inflammatory cytokine. SPRC administered 12 h before AP induction did not cause significant improvement in pancreatic and lung inflammation. Plasma H_2_S concentration showed significant difference in H_2_S levels between control, vehicle and SPRC (administered 3 h before AP) treatment groups. In conclusion, these data provide evidence for protective effects of SPRC in AP possibly by virtue of its slow release of endogenous H_2_S.

## Introduction

Hydrogen sulfide (H_2_S), a novel endogenous gaseous mediator, has been explored recently for its physiological and pathological roles. H_2_S is synthesized from L-cysteine, a sulfur-containing amino acid substrate [1], facilitated by the two key enzymes Cystathionine γ-lyase (CSE, EC4.4.1.1) and cystathionine β-synthase (CBS, EC4.2.1.22). Numerous animal studies have shown the beneficial effects of H_2_S, especially in cardiovascular and neurological disorders [2]. But the role of H_2_S in inflammation is only recently beginning to emerge and the exact role of H_2_S in inflammation is still not clearly understood. While pro-inflammatory effects of H_2_S were observed in various models of inflammation, some studies have also reported anti-inflammatory effects of H_2_S [3]–[5]. In the former studies, plasma H_2_S level, tissue H_2_S synthesizing enzyme activity and CSE expression were increased in inflammation and inhibition of H_2_S synthesis by a CSE inhibitor reduced the inflammation [6]–[11]. However, treatments with either H_2_S-releasing non steroidal anti-inflammatory drugs (e.g. s-diclofenac, ATB-429) or H_2_S donors (e.g. sodium hydrosulfide (NaHS), Lawesson's reagent, N-acetylcysteine, GYY4137) have also reported anti-inflammatory activity in inflammation [12]–[18]. Thus in addition to blocking endogenous H_2_S in inflammation, several studies are also in progress to develop sustained-releasing H_2_S donors to combat inflammation more effectively.

Acute pancreatitis (AP) is an acute inflammatory disorder of pancreas. It is potentially lethal and the incidence of AP has been increasing over recent years. Approximately 20–25% of the patients suffers a severe attack, and 30–50% of these will die [19], [20]. Although the mortality rate following pancreatitis has significantly improved over the past few decades, treatment options currently available are limited, and predominantly aimed at supportive therapy. Several pharmacological agents have been studied in animal models of AP and in clinical settings of pancreatitis, with variable success [19], [21].

Our group has previously shown the anti-inflammatory effects of low doses of NaHS in mouse model of AP [22]. However due to the narrow therapeutic window and potentially toxic effects associated with high doses of NaHS, researchers are focusing on developing novel H_2_S donors [22]. H_2_S-releasing non-steroidal anti-inflammatory drugs (NSAID) like ACS15 and ATB-429 and other drugs like GYY4137 have demonstrated anti-inflammatory effects *in vivo*/*vitro* studies [12]–[16]. S-propargyl-cysteine (SPRC) is a structural analog of S-allyl cysteine (SAC) with a common cysteine-containing structure [23]. SAC, a water-soluble organosulfur compound of aged garlic extract (AGE), is well known for its cardio-protective and anti-cancer effects [24]. Research studies have shown protective effects of SAC against carbon tetrachloride-induced oxidative stress and pulmonary fibrosis and carbon tetrachloride-induced acute liver injury in rats [25], [26]. SAC has been shown to be a substrate of CSE and also induce CSE expression resulting in an increase in H_2_S production [27]. Interestingly, SPRC has been reported to show protective effects against myocardial infarctions in both adult rat hearts and neonatal cardiomyocytes through H_2_S pathway [23]. These novel cysteine containing compounds (SPRC, SAC and S-propyl-L-cysteine (SPC)) have shown to reduce the deleterious effects of oxidative stress in rat acute myocardial infarction [28].

In this study, we aimed to investigate the effects of SPRC in caerulein-induced AP in mice to test the hypothesis that SPRC could be beneficial against AP. In addition to the evaluation of inflammation in the pancreas and lung, we also determined the plasma H_2_S levels with SPRC treatment.

## Materials and Methods

### Experimental procedures

All animal experiments were approved by the Animal Ethics Committees of National University of Singapore, Singapore (permit number 807/05) and University of Otago (permit number CET1/10) and were carried out in accordance with established International Guiding Principles for Animal Research. Swiss mice (male, 20–25 g) were used and maintained in the Animal Housing Unit in an environment with controlled temperature (21–24°C) and lighting (12 h light-dark cycle). Standard laboratory chow and drinking water were provided *ad libitum*. A period of at least 2 days was allowed for the animals to acclimatize before any experimental procedures were undertaken.

### Induction of AP

Caerulein was obtained from Bachem (Bubendorf, Switzerland). SPRC was synthesized by Dr Zhu Yi Zhun. Mice were randomly assigned to four groups (n = 10 per group). Group 1: Animals were given hourly intraperitoneal (i.p.) injections of normal saline (CTRL group). Group 2: Animals were treated with distilled water (DW) followed by hourly i.p. injections of caerulein (50 µg/kg) over 10 h to induce AP (Veh+Cae). Group 3: Animals were treated with SPRC (10 mg/kg), 3 h before hourly injections of caerulein (50 µg/kg) over 10 h (SPRC 3 h+Cae). Group 4: Animals were treated with SPRC (10 mg/kg), 12 h before hourly injections of caerulein (50 µg/kg) over 10 h (SPRC 12 h+Cae). SPRC was dissolved in DW. One hour after the last caerulein injection animals were sacrificed by an i.p. injection of a lethal dose of pentobarbital (50 mg/kg: Nembutal, CEVA Sante Animale, Naaldwijk, Netherlands). Blood, pancreas and lung tissues were collected. Samples of pancreas and lung were weighed, snap frozen in liquid nitrogen and then stored at −80°C for subsequent measurement of tissue myeloperoxidase (MPO) activities, and cytokines as described in detail below. Harvested heparinized blood was centrifuged (10,000 rpm, 10 min, 4°C) and the plasma was aspirated and stored at −80°C for subsequent detection of plasma amylase and H_2_S. Parts of the pancreas and lung were also fixed in 10% v/v neutral phosphate-buffered formalin for more than 48 h and then were processed for histology.

### Amylase estimation

Plasma amylase activity was measured using a kinetic spectrophotometric assay. Plasma samples were incubated with the Amylase reagent (Sigma, St. Louis, Mo) for 2 min at 37°C, and absorbance was measured every min for the subsequent 2 min at 405 nm using manufacturer's instructions [7], [13]. The resulting change in absorbance was used to calculate the amylase activity.

### MPO estimation

Sequestration of inflammatory cells in pancreas and lung were quantified by measuring tissue MPO activity [7], [13]. Tissue samples were thawed, homogenized in 20 mM phosphate buffer (pH 7.4), centrifuged (13,000 rpm, 10 min, 4°C), and the resulting pellet resuspended in 50 mM phosphate buffer (pH 6.0) containing 0.5% w/v hexadecyltrimethylammonium bromide (Sigma). The suspension was subjected to four cycles of freezing and thawing and further disrupted by sonication (40 s). The sample was then centrifuged (13,000 rpm, 5 min, 4°C), and the supernatant was used for the MPO assay. The sample was mixed with equal volume of 1-component tetramethylbenzidine (TMB) substrate (Sureblue), incubated for a fixed time, and then terminated by adding equal volume of 2N H_2_SO_4_. The absorbance was measured at 450 nm and corrected for the calculated DNA of the tissue sample [29]. Results were expressed as enzyme activity (fold increase over corresponding saline injected control groups).

### Morphological examination

Paraffin-embedded pancreas and lung samples were sectioned (5 µm), stained with hematoxylin/eosin (H and E) and were examined with light microscopy by an experienced observer blinded with respect to different experimental groups. Acinar-cell injury/necrosis was quantitated by morphometry as described [7]. For these studies, 10 randomly chosen microscopic fields were examined for each tissue sample and the extent of acinar-cell injury/necrosis was expressed as the percent of the total acinar tissue that was occupied by areas meeting the criteria for injury/necrosis. Those criteria were defined as either (*i*) the presence of acinar-cell ghosts or (*ii*) vacuolization and swelling of acinar cells and the destruction of the histoarchitecture of whole or parts of the acini, both of which had to be associated with an inflammatory reaction. Capillary-alveolar membrane thickness was visually estimated [29] on a scale ranging from 0 to 4, with 0 being the thinnest and 4 the thickest as observed in the course of these experiments.

### Enzyme-linked immunosorbent assay (ELISA) of cytokines

The levels of cytokines (IL-1β, IL-6, IL-10 and TNF-α) were measured in pancreas and lung tissue homogenate by a sandwich ELISA using DuoSet ELISA kits. Briefly, anti-cytokine primary antibodies were coated onto 96-well ELISA plates and incubated overnight at room temperature. Samples and standards were added to the wells and incubated for 2 h, the wells were washed, and a biotinylated goat anti-mouse cytokines antibodies were added for 2 h. Plates were washed again, and streptavidin antibodies conjugated to HRP were added for 20 min. After a further wash, TMB was added for color development, and the reaction was terminated with 2 N H_2_SO_4_. Absorbance was measured at 450 nm. Cytokine concentrations of samples were estimated from the standard curve. The cytokine concentration was then corrected for the DNA content of the tissue [30].

### Measurement of plasma H_2_S

Aliquots (300 µl) of plasma were mixed with distilled water (250 µl), zinc acetate (1% w/v, 150 µl), N, N-dimethyl-p-phenylenediamine sulphate (20 µM; 100 µl) in 7.2 M HCl and FeCl_3_ (30 µM; 100 µl) in 1.2 M HCl. After 10 min incubation, trichloroacetic acid (10% w/v, 300 µl) was added to the above mixture to stop the reaction. Samples were centrifuged (5,000 rpm, 5 min) and then the supernatant (300 µl) was added into 96- well plates. The absorbance was measured by microplate reader (SPECTRAFluor Plus, Tecan) at 670 nm. All samples were assayed in duplicate and H_2_S was calculated using a calibration curve of NaHS (3.12–200 µM). The plasma H_2_S concentrations were expressed as µM.

### Statistical analysis

Data were expressed as the mean ± standard error of the mean (s.e.m.). In all figures, vertical error bars denote the s.e.m. The significance of differences between groups was evaluated by using analysis of variance (ANOVA) when comparing 3 or more groups. Tukey and/or LSD method were used as a post hoc test for comparison among different groups. A *P* value of <0.05 was considered to indicate a significant difference.

## Results

### Effect of SPRC on plasma amylase in caerulein-induced AP

**Figure 1:**
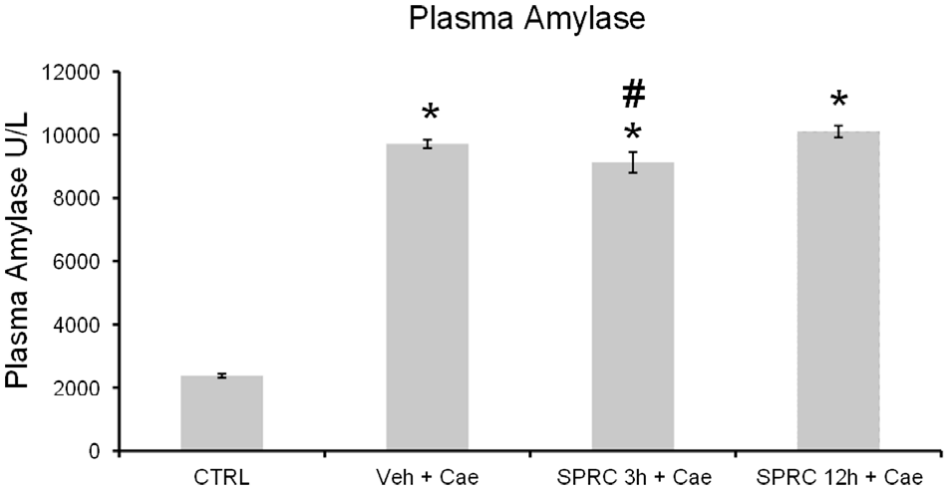
Effect of SPRC treatment on plasma amylase activity. Acute pancreatitis was induced by intraperitoneal (i.p.) administration of caerulein (50 µg/kg, hourly for 10 h). Mice were pre-treated with vehicle or SPRC (10 mg/kg, i.p.), either 3 h or 12 h before the induction of pancreatitis. One hour after the last caerulein injection, mice were killed by a lethal dose of pentobarbitone (50 mg/kg, i.p.), and plasma amylase activity was measured as described in “Materials and methods”. Key: CTRL, animals injected only with saline (i.p., hourly for 10 h) without the induction of pancreatitis; Veh+Cae, pre-treatment with vehicle (distilled water) before the induction of pancreatitis; SPRC 3 h+Cae, SPRC injected 3 h before the induction of pancreatitis; SPRC 12 h+Cae, SPRC injected 12 h before the induction of pancreatitis. Results shown are the mean ± s.e.m., n = 10 mice per group. ^*^ *P*<0.05 when compared to the CTRL group; ^#^ *P*<0.05 when compared to the Veh+Cae group. CTRL, control; Cae, caerulein; Veh, vehicle; SPRC, S-propargyl-cysteine.

The evidence of pancreatic injury induced by caerulein is generally confirmed by an increase in plasma amylase. Thus the effect of SPRC treatment on plasma amylase in AP was evaluated, especially in comparison with the corresponding values in saline and vehicle pretreated mice. As shown in Fig. 1, mice pretreated with vehicle or SPRC followed by hourly caerulein injections, pancreatitis was manifested by significant rise in plasma amylase activity compared to the mice injected with hourly saline only (*P*<0.05). However among the SPRC treatment groups, plasma amylase was significantly reduced only in mice injected with SPRC 3 h before the induction of pancreatitis compared to vehicle pretreated mice (P<0.05).

### Effect of SPRC on pancreas MPO and histology in caerulein-induced AP

**Figure 2:**
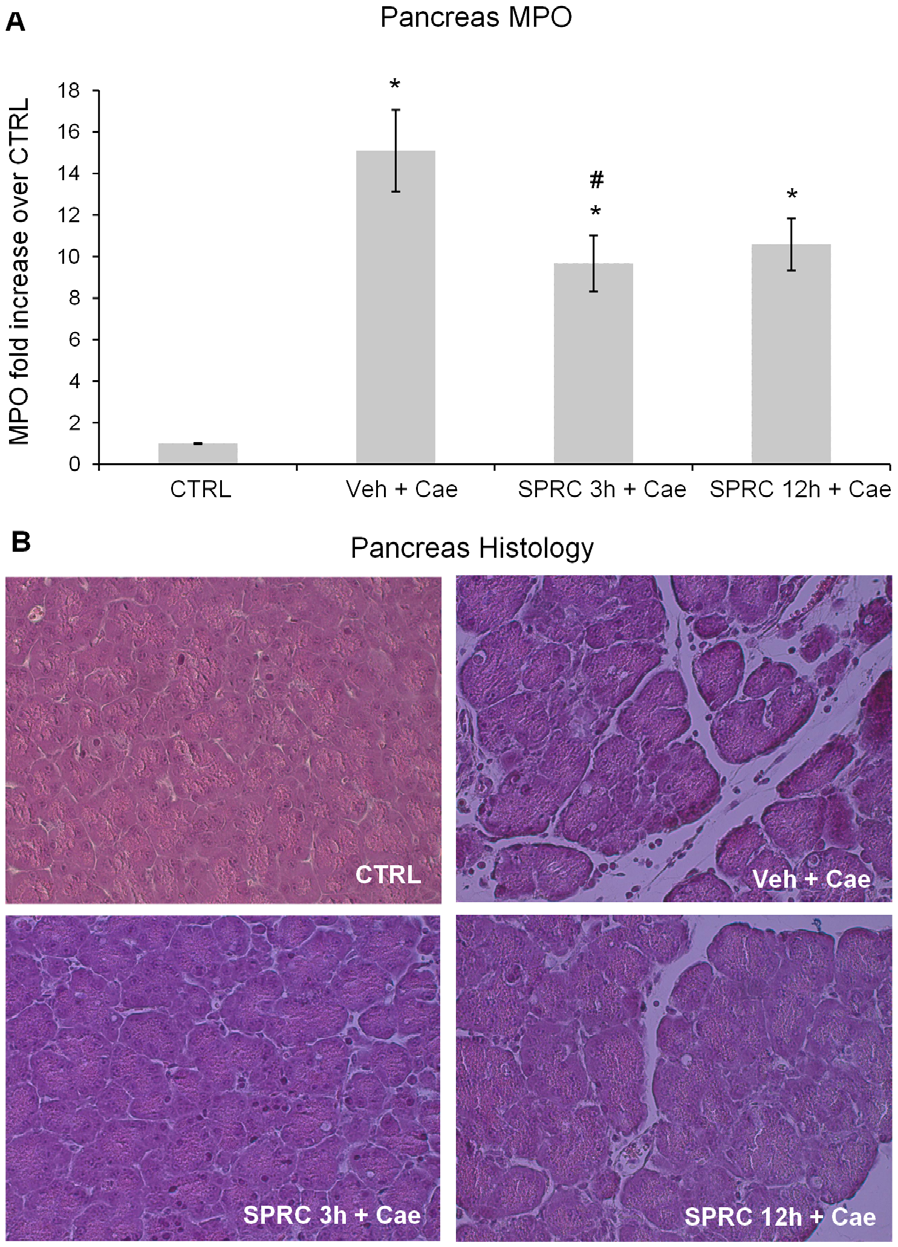
Effect of SPRC treatment on pancreatic inflammation. **A.** myeloperoxidase (MPO) activity. **B.** histology. Acute pancreatitis was induced by intraperitoneal (i.p.) administration of caerulein (50 µg/kg, hourly for 10 h). Mice were pre-treated with vehicle or SPRC (10 mg/kg, i.p.), either 3 h or 12 h before the induction of pancreatitis. One hour after the last caerulein injection, mice were killed by a lethal dose of pentobarbitone (50 mg/kg, i.p.) and pancreatic MPO activity and histology were evaluated as described in “Materials and methods”. Key: CTRL, animals injected only with saline (i.p., hourly for 10 h) without the induction of pancreatitis; Veh+Cae, pre-treatment with vehicle (distilled water) before the induction of pancreatitis; SPRC 3 h+Cae, SPRC injected 3 h before the induction of pancreatitis; SPRC 12 h+Cae, SPRC injected 12 h before the induction of pancreatitis. Results shown are the mean ± s.e.m., n = 10 mice per group. Arrow shows oedema and infiltration of inflammatory cells. ^*^ *P*<0.05 when compared to the CTRL group; ^#^ *P*<0.05 when compared to the Veh+Cae group. CTRL, control; Cae, caerulein; Veh, vehicle; SPRC, S-propargyl-cysteine.

Pancreatic inflammation was assessed by measuring pancreatic MPO activity and histology. Measurement of MPO enzyme which is located in azurophile granules of neutrophils and monocytes reflects inflammatory cell infiltration in tissue. There was a significantly elevated MPO in mice administered with vehicle or SPRC compared to saline (CTRL) group (Fig. 2A). However within the SPRC treated groups, mice injected with SPRC 3 h before the induction of pancreatitis showed significant reduction in MPO activity as compared to vehicle treated mice (Fig. 2A). Histological examination of sections of pancreas of vehicle pretreated mice showed evidence of oedema, destruction of histoarchitecture of the acini and infiltration of inflammatory cells (Fig. 2B, Veh+Cae). However, among the SPRC groups, mice given SPRC 3 h before AP induction showed a significantly reduced pancreatic edema and inflammatory cell infiltration compared to the vehicle pretreated mice (Fig. 2B, SPRC 3 h+Cae). Quantitation of acinar cell injury/necrosis showed that Veh+Cae treated mice had 42±3.6% acinar cell injury/necrosis, compared with 1±0.5% for Control, 10±0.9% for SPRC 3 h+Cae, and 14±1.6% for SPRC 12 h+Cae.

### Effect of SPRC on AP-associated lung injury

**Figure 3:**
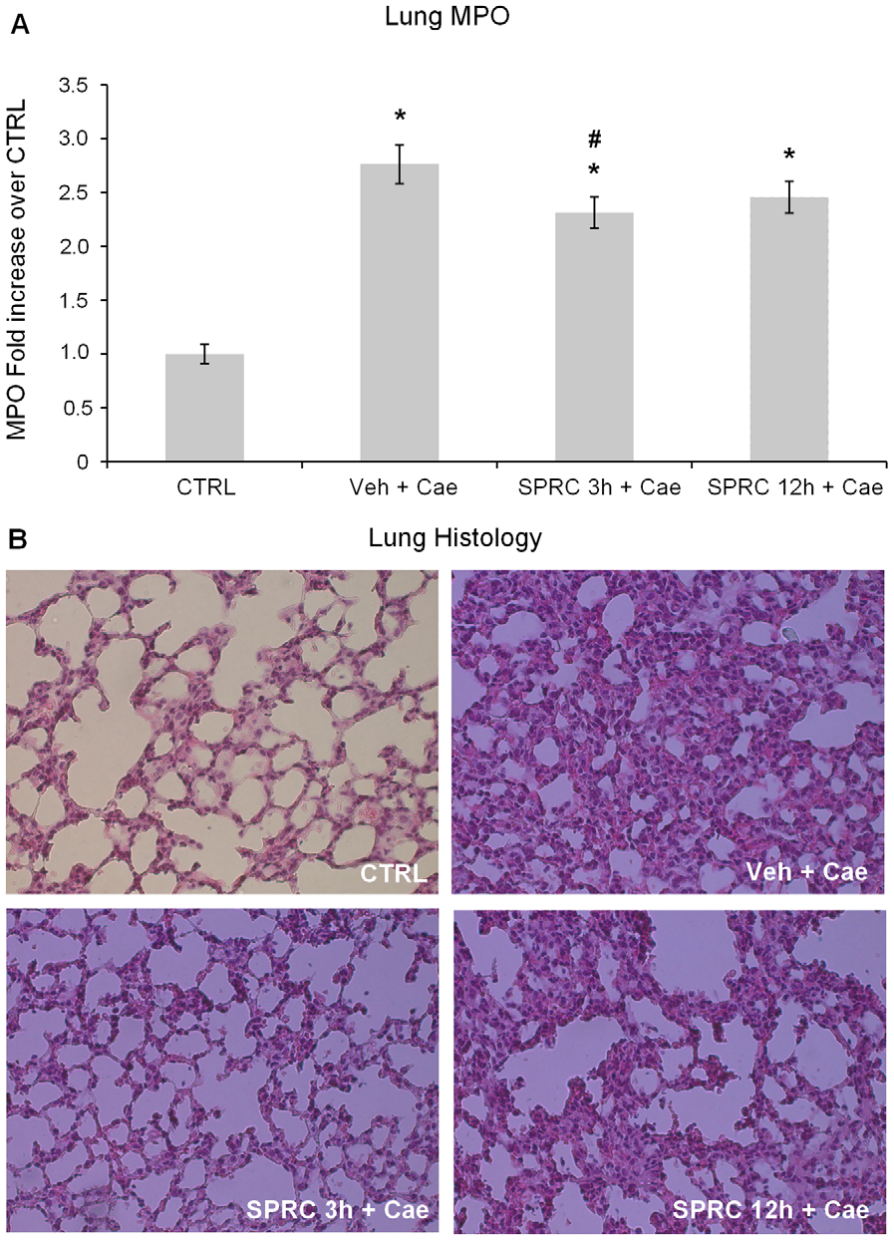
Effect of SPRC on pancreatitis-associated lung injury. **A.** myeloperoxidase (MPO) activity. **B.** histology. Acute pancreatitis was induced by intraperitoneal (i.p.) administration of caerulein (50 µg/kg, hourly for 10 h). Mice were pre-treated with vehicle or SPRC (10 mg/kg, i.p.), either 3 h or 12 h before the induction of pancreatitis. One hour after the last caerulein injection, mice were killed by a lethal dose of pentobarbitone (50 mg/kg, i.p.) and lung MPO activity and histology were evaluated as described in “Materials and methods”. Key: CTRL, animals injected only with saline (i.p., hourly for 10 h) without the induction of pancreatitis; Veh+Cae, pre-treatment with vehicle (distilled water) before the induction of pancreatitis; SPRC 3 h+Cae, SPRC injected 3 h before the induction of pancreatitis; SPRC 12 h+Cae, SPRC injected 12 h before the induction of pancreatitis. Results shown are the mean ± s.e.m., n = 10 mice per group. ^*^ *P*<0.05 when compared to the CTRL group; ^#^ *P*<0.05 when compared to the Veh+Cae group. CTRL, control; Cae, caerulein; Veh, vehicle; SPRC, S-propargyl-cysteine.

Consistent with our earlier studies, AP induced by 10 hourly injections of caerulein (50 µg/kg) was associated with lung injury. As shown in Fig. 3A, caerulein-induced AP was associated with a significant rise in lung MPO activity compared to saline (CTRL) group. Histological examination of the lung sections further confirmed lung injury in AP as evidenced by alveolar thickening and abundance of inflammatory cell infiltration (Fig. 3B, Veh+Cae). However, mice treated with SPRC 3 h before the induction of pancreatitis had significant reduction in cellular infiltration as evidenced by decreased lung MPO activity (Fig. 3A, SPRC 3 h+Cae) and histology (Fig. 3B, SPRC 3 h+Cae). Similar protection was not seen in mice administered SPRC 12 h before the induction of pancreatitis (Fig. 3A and Fig. 3B, SPRC 12 h+Cae). Quantitation of alveolar thickening (on a score of 0–4), an evidence of lung injury, showed that Veh+Cae treated mice had 3.5±0.3 alveolar thickening, compared with 0.38±0.1 for Control, 1.5±0.3 for SPRC 3 h+Cae, and 3.5±0.3 for SPRC 12 h+Cae. Thus, treatment with SPRC 10 mg/kg given 3 h before the induction of pancreatitis resulted in a marked reduction in the severity of pancreatitis-associated lung injury.

### Effect of SPRC on pancreatic and pulmonary cytokines

**Figure 4:**
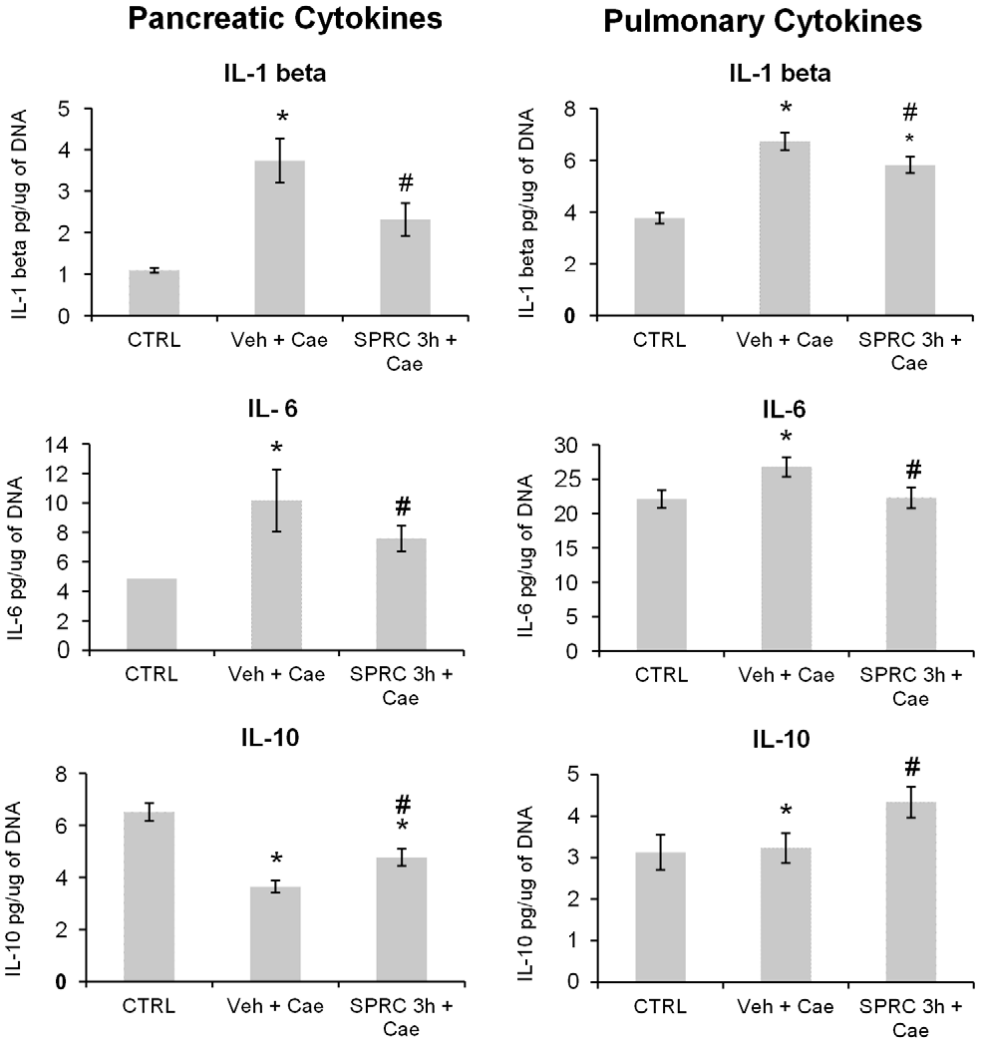
Effect of SPRC on pancreatic and pulmonary cytokines. Acute pancreatitis was induced by intraperitoneal (i.p.) administration of caerulein (50 µg/kg, hourly for 10 h). Mice were pre-treated with vehicle or SPRC (10 mg/kg, i.p.) 3 h before the induction of pancreatitis. One hour after the last caerulein injection, mice were killed by a lethal dose of pentobarbitone (50 mg/kg, i.p.) and pancreatic and lung cytokine levels were analyzed as described in “Materials and methods”. Key: CTRL, animals injected only with saline (i.p., hourly for 10 h) without the induction of pancreatitis; Veh+Cae, pre-treatment with vehicle (distilled water) before the induction of pancreatitis; SPRC 3 h+Cae, SPRC injected 3 h before the induction of pancreatitis. Results shown are the mean ± s.e.m., n = 8–10 mice per group. ^*^ *P*<0.05 when compared to the CTRL group; ^#^ *P*<0.05 when compared to the Veh+Cae group. CTRL, control; Cae, caerulein; Veh, vehicle; SPRC, S-propargyl-cysteine.

Cytokines, one of the major inflammatory mediators, play a critical role in the pathogenesis of pancreatitis and more so in the subsequent inflammatory damage. Based on our initial data with SPRC treatment given at different time points before the induction of pancreatitis, we decided to see if the reduction in pancreatic and pulmonary inflammation with SPRC administered 3 h prior to AP had any effect on cytokine levels in pancreas and lung. As expected, pro-inflammatory cytokines (IL-1β and IL-6) were significantly increased in pancreas as well as lung tissue in vehicle treated group compared to saline control animals (Fig. 4, upper and middle panels). Interestingly, these pro-inflammatory cytokines were significantly reduced in mice pretreated with SPRC 3 h before the induction of pancreatitis (Fig. 4, upper and middle panels).

Pancreatic anti-inflammatory cytokine IL-10 was significantly lowered in vehicle treated group compared to saline control mice but this trend was not observed in the corresponding lungs (Fig. 4, lower panel). However, SPRC treatment 3 h before the induction of pancreatitis significantly increased both pancreatic and lung IL-10 levels compared to the corresponding values in vehicle treated group (Fig. 4, lower panel).

### Effect of SPRC on plasma H_2_S concentration

**Figure 5:**
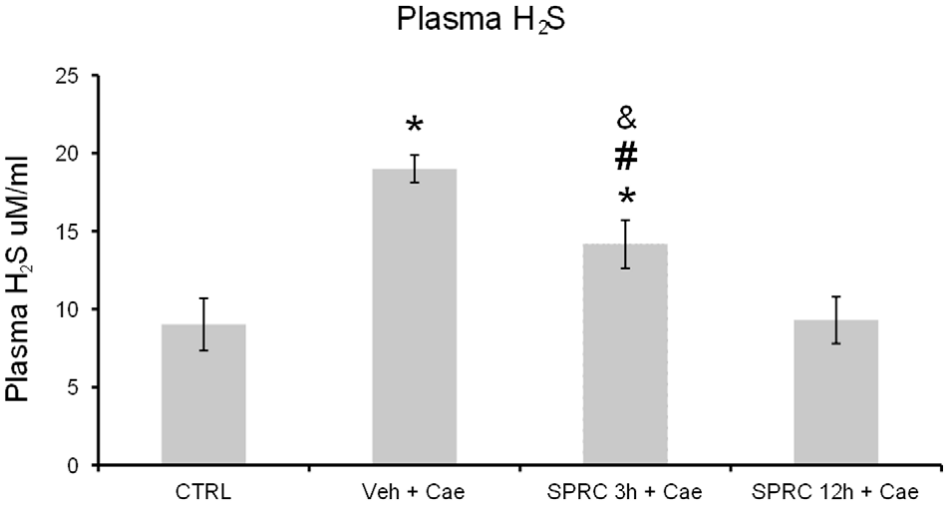
Effect of SPRC on plasma H_2_S concentration. Acute pancreatitis was induced by intraperitoneal (i.p.) administration of caerulein (50 µg/kg, hourly for 10 h). Mice were pre-treated with vehicle or SPRC (10 mg/kg, i.p.), 3 h before the induction of pancreatitis. One hour after the last caerulein injection, mice were killed by a lethal dose of pentobarbitone (50 mg/kg, i.p.) and plasma H_2_S level was analyzed as described in “Materials and methods”. Key: CTRL, animals injected only with saline (i.p., hourly for 10 h) without the induction of pancreatitis; Veh+Cae, pre-treatment with vehicle (distilled water) before the induction of pancreatitis; SPRC 3 h+Cae, SPRC injected 3 h before the induction of pancreatitis. Results shown are the mean ± s.e.m., n = 8–10 mice per group. ^*^ *P*<0.05 when compared to the CTRL group; ^#^ *P*<0.05 when compared to the Veh+Cae group. CTRL, control; Cae, caerulein; Veh, vehicle; SPRC, S-propargyl-cysteine.

Plasma H_2_S concentration was measured 1 h after the last injection of saline/caerulein. There was a significant increase in plasma H_2_S concentration in the AP mice pretreated with either vehicle or SPRC (injected 3 h before AP) compared to saline control group (Fig. 5). However among the mice subjected to AP, plasma H_2_S concentration was significantly lower in mice pretreated with SPRC (injected 3 h before AP) compared to vehicle treated group (Fig. 5).

## Discussion

SPRC is a newly synthesized H_2_S donor and is a structural analog of SAC. We aimed to investigate the therapeutic potential of SPRC in caerulein-induced AP. SPRC was injected at two different time points, either 3 or 12 h before the induction of AP. Results showed that pancreatic injury, as evidenced by plasma amylase, pancreatic MPO and histology and pulmonary injury, as evidenced by lung MPO and histology were significantly ameliorated in the mice treated with SPRC 3 h prior to the induction of pancreatitis. Administration of SPRC 12 h before AP induction, on the other hand, was not very effective in protecting against AP and associated lung injury, possibly because the therapeutic window was missed. In addition we evaluated if the improvement in pancreatic and pulmonary inflammation with SPRC given 3 h before induction of AP had any effects on pancreas and lung cytokine levels. Cytokines play a critical role in the early pathophysiological events of the disease by recruiting more inflammatory cells from the peripheral blood to the site of injury [31]–[33]. A recent clinical study showed a clear relationship between MPO, severity of AP and the blood cytokine levels [34]. Prolonged increase in MPO activity is reported to indicate continued neutrophil activation, with the liberation of cytokines and other biologically active substances like reactive oxygen species [34]. We observed a significant reduction in the pro-inflammatory cytokines (IL-1β and IL-6) in pancreas and lung of mice treated with SPRC 3 h before the induction of AP compared to the mice pretreated with vehicle.

Pancreatic and pulmonary anti-inflammatory cytokine Interleukin-10 (IL-10) levels were significantly increased in mice pretreated with SPRC compared to vehicle treated group. IL-10 is a potent inhibitor of cytokines and has been shown to attenuate pancreatitis in animal models. Systemic administration of adenoviral vectors expressing IL-10 significantly reduced the degree of pancreatic and liver injury in mice models of acute necrotizing pancreatitis [35]. However, IL-10 therapy in human for prevention of AP after post-endoscopic retrograde cholangiopancreatography is still controversial [36], [37]. Collectively, our results demonstrated that SPRC injected 3 h prior to the induction of AP showed a beneficial effect on pancreatic and lung injury along with modulating inflammatory cytokines.

Furthermore, we investigated if SPRC treatment affected plasma H_2_S levels in AP and the mechanism by which inflammation was modulated by SPRC. SPRC, like SAC, could potentially influence the synthesis of endogenous H_2_S. *In vitro*, SPRC was found to increase H_2_S synthesizing enzyme activity in normal pancreatic acini compared to the vehicle control, but the difference was not significant in the presence of caerulein hyper-stimulation (unpublished data). However, while we observed a significant increase in plasma H_2_S concentration in AP-induced mice treated with vehicle, SPRC injected 3 h before AP lowered this increase. As expected, high plasma H_2_S concentration observed in vehicle treated mice after induction of AP was pro-inflammatory and damaging. Administration of SPRC 3 h before AP induction and thus the slow release of H_2_S could have inhibited CSE by a feedback mechanism resulting in significantly lower levels of H_2_S compared to the vehicle treated mice. Administration of a slow H_2_S releasing compound has previously been shown to be associated with a decrease in endogenous H_2_S formation [14]. Evidently, further studies are needed to explore the mechanisms of SPRC effects in AP in detail.

In conclusion, SPRC 10 mg/kg injected 3 h prior to induction of AP ameliorated the disease by reducing the inflammatory cell infiltration in pancreas and lung and by modulating pro- and anti-inflammatory cytokine profile in plasma. Thus SPRC provides a valuable lead for the treatment of AP. It could be postulated that the beneficial effects of SPRC in AP could be by virtue of its slow release of endogenous H_2_S and a possible negative feedback mechanism on CSE.
